# Autophagy-mediated occludin degradation contributes to blood–brain barrier disruption during ischemia in bEnd.3 brain endothelial cells and rat ischemic stroke models

**DOI:** 10.1186/s12987-020-00182-8

**Published:** 2020-03-14

**Authors:** Kyeong-A Kim, Donghyun Kim, Jeong-Hyeon Kim, Young-Jun Shin, Eun-Sun Kim, Muhammad Akram, Eun-Hye Kim, Arshad Majid, Seung-Hoon Baek, Ok-Nam Bae

**Affiliations:** 1 College of Pharmacy Institute of Pharmaceutical Science and Technology, Hanyang University, Ansan, Republic of Korea; 2 Faculty of Pharmacy, University of Sindh, Jamshoro, Pakistan; 3 Sheffield Institute for Translational Neuroscience, University of Sheffield, Sheffield, England, UK; 4 College of Pharmacy and Research Institute of Pharmaceutical Science and Technology (RIPST), Ajou University, Suwon, Republic of Korea

**Keywords:** Ischemic stroke, Oxygen–glucose deprivation (OGD), Middle cerebral artery occlusion (MCAO), Occludin, Blood–brain barrier (BBB), Autophagy

## Abstract

**Background:**

The blood–brain barrier (BBB) maintains homeostasis of the brain environment by tightly regulating the entry of substances from systemic circulation. A breach in the BBB results in increased permeability to potentially toxic substances and is an important contributor to amplification of ischemic brain damage. The precise molecular pathways that result in impairment of BBB integrity remain to be elucidated. Autophagy is a degradation pathway that clears damaged or unnecessary proteins from cells. However, excessive autophagy can lead to cellular dysfunction and death under pathological conditions.

**Methods:**

In this study, we investigated whether autophagy is involved in BBB disruption in ischemia, using in vitro cells and in vivo rat models. We used brain endothelial bEnd.3 cells and oxygen glucose deprivation (OGD) to simulate ischemia in culture, along with a rat ischemic stroke model to evaluate the role of autophagy in BBB disruption during cerebral ischemia.

**Results:**

OGD 18 h induced cellular dysfunction, and increased permeability with degradation of occludin and activation of autophagy pathways in brain endothelial cells. Immunostaining revealed that occludin degradation is co-localized with ischemic autophagosomes. OGD-induced occludin degradation and permeability changes were significantly decreased by inhibition of autophagy using 3-methyladenine (3-MA). Enhanced autophagic activity and loss of occludin were also observed in brain capillaries isolated from rats with middle cerebral artery occlusion (MCAO). Intravenous administration of 3-MA inhibited these molecular changes in brain capillaries, and recovered the increased permeability as determined using Evans blue.

**Conclusions:**

Our findings provide evidence that autophagy plays an important role in ischemia-induced occludin degradation and loss of BBB integrity.

## Introduction

A well-controlled physiological environment is required for normal brain function [1], and the entry of substances or cells circulating in the blood is strictly restricted by blood–brain barrier (BBB) [2]. Tight junctions (TJs) between brain endothelial cells are major structural and functional components of the BBB and play a crucial role in sealing the extracellular space. Damage to or loss of TJs results in a compromised BBB [3, 4]. TJs are mainly composed of transmembrane proteins such as claudins, occludin, and intracellular accessory proteins such as zonula occludens (ZOs) [5, 6]. Each TJ component has a specific role and provides a vital and important contribution to overall BBB integrity [7–11]. In particular, occludin enhances the stability of the TJ assembly which assists in their function as barriers [12, 13]. Several studies have reported that the alteration or decrease in occludin during cerebral ischemia results in increased BBB permeability [14, 15]. Although matrix metalloproteinase (MMP)-mediated occludin degradation has been reported as a potential pathway for TJ dysfunction, the exact molecular mechanisms underlying ischemic BBB disruption remain to be elucidated.

Autophagy is constantly occurring at a basal level in cells and is the mechanism by which organelles or proteins are degraded and recycled [15]. Autophagy involves the formation of membrane bound intracellular vacuoles called autophagosomes, which engulf the target protein or organelle [16]. These autophagosomes then fuse with intracellular lysosomes which digest target proteins by lysosomal enzymes [17]. While this pathway is a normal maintenance and recycling mechanism, recent data have suggested that autophagy also occurs during pathological conditions in which high levels of autophagy contribute to cellular dysfunction and death [18, 19]. We have recently reported on the excessive activation of autophagic activity in brain tissue during ischemic stroke in rats, and found that ischemia-induced autophagy contributed to neuronal cell death [18]. However, little is known about whether autophagy is involved in BBB disruption during ischemia.

In the present study, we used cultured brain endothelial cells and a rat middle cerebral artery occlusion model to investigate whether autophagy is involved in BBB dysfunction during ischemia. In particular, we investigated whether autophagy plays a role in injury to occludin.

## Materials and methods

### Materials

Silicone-coated 4-0 monofilament nylon sutures were purchased from Doccol Co. (Redlands, CA, USA). Primary antibodies against lysosomal-associated membrane protein 1 (LAMP-1), and p62/SQSTM1, and horseradish peroxidase (HRP)-conjugated secondary antibodies were purchased from Santa Cruz Biotechnology (Dallas, TX, USA). Primary antibodies against LC3B and β-actin were obtained from Sigma-Aldrich (St. Louis, MO, USA). A primary antibody against CD31 was obtained from R&D Systems (Minneapolis, MN, USA). A primary antibody against occludin and the fluorescently labeled secondary antibodies of Alexa Fluor 488 donkey anti-mouse and Alexa Fluor 555 donkey anti-rabbit were purchased from Thermo Fisher Scientific (Rockford, IL, USA). All other chemicals were purchased from Sigma-Aldrich.

### Cell culture

The bEnd.3 cell line, immortalized mouse brain endothelial cells, was obtained from American Type Culture Collection (Manassas, VA, USA). bEnd.3 cells were grown in DMEM (Dulbecco’s Modified Eagle’s Medium with 4500 mg/L d-glucose, 110 mg/L sodium pyruvate, 1.5 g/L sodium bicarbonate, and l-glutamine; Welgene, Daegu, Korea) supplemented with 10% fetal bovine serum (FBS; Mediatech Inc., Manassas, VA, USA), 100 units/mL of penicillin, and 100 μg/mL of streptomycin (Welgene, Korea). bEnd.3 cells were maintained in a humidified incubator at 37 °C with 5% CO_2_ and 95% air. All experiments were carried out when the density of cells was 90–100%.

### Oxygen–glucose deprivation (OGD) exposure

bEnd.3 cells were rinsed twice with Earle’s Balanced Salt Solution (EBSS; 116 mM NaCl, 5.4 mM KCL, 26.2 mM NaHCO_3_, 1.8 mM CaCl_2_, 1 mM NaH_2_PO_4_H_2_O, 0.8 mM MgSO_4_, and 0.01 mM glycine), and the media were replaced with glucose-free EBSS or EBSS supplemented with 5.5 mM glucose for OGD stimulation or control treatment, respectively. Cell plates were placed in a hypoxia chamber (Billups-Rothenberg Inc., San Diego, CA, USA), and the air was replaced with OGD gas (95% N_2_ and 5% CO_2_) by flushing. Cells were exposed to the OGD condition for 6, 12 or 18 h at 37 °C to measure the cell viability. After selecting 18 h OGD as the ischemic insult, cells were exposed to 18 h OGD for the other experiments. Oxygen depletion in the chamber was monitored using BD GasPak™ Dry Anaerobic Indicator Strips (BD, Franklin Lakes, NJ, USA). For autophagic inhibition experiments, bEnd.3 cells were treated with 3-methyladenine (3-MA; Sigma-Aldrich) for 1 h prior to OGD stimulation. The concentration of 3-MA was selected according to the previous reports [16, 20]. Treatment with 3-MA was continued during OGD exposure.

### Measurement of cell viability

bEnd.3 cells were seeded on 48- or 96-well plates and incubated to confluence. To determine the effect of OGD on cell viability, cells were exposed to OGD for 18 h and then incubated with 0.5 mg/mL MTT for 2 h [21]. The supernatants were discarded and insoluble formazan was dissolved using dimethyl sulfoxide. The absorbance of the solution was measured at 570 nm using an EnSpire microplate reader (PerkinElmer, Santa Clara, CA, USA).

### Determination of in vitro permeability

bEnd.3 cells were seeded on 0.4-µm Pore Polycarbonate Membrane Inserts in 6.5-mm Transwell^®^ plates (Corning, New York, NY, USA) at a density of 2 × 10^4^ cells/well and grown for 6 days to confluence. After OGD exposure, permeability was determined by FITC-dextran or transendothelial electrical resistance (TEER). For the FITC-dextran permeability assay, 20 µg/mL FITC-dextran (molecular weight 70 kDa, Sigma-Aldrich) in phosphate buffered saline (PBS) was added to the upper compartment while the lower compartment was filled with fresh PBS after OGD exposure. After incubation for 30 min in the dark, the transferred amount of FITC-dextran in the lower compartment, which passed through the membrane insert via damaged junctional integrity, was measured by an EnSpire microplate reader at 490-nm excitation and 520-nm emission wavelength. In measurement of TEER, which indicates the integrity of the tight junction, the resistance was measured before and right after OGD exposure using electronic circuit of EVOM2 and STX2 electrode (World Precision Instruments, Sarasota, FL, USA). All TEER data was subtracted by the value from the cell-free inserts of each treatment group.

### Immunofluorescence staining

Cells were seeded at a density of 2 × 10^4^ cells/well into a Lab-Tek™ 8-well Chambered Coverglass (Thermo Fisher Scientific, Rochester, NY, USA). When the cells reached confluence, cells were exposed to OGD for 18 h. Cells were fixed with 4% paraformaldehyde for 15 min, permeabilized with 0.5% Triton X-100 (Promega, Madison, WI, USA) for 10 min, and blocked using 1% BSA (Sigma-Aldrich) in PBS. The cells were incubated with primary antibodies (anti-LC3, anti-occludin or anti-LAMP1 antibodies) diluted in 1% BSA and then further incubated with secondary antibodies (Alexa Fluor 555-conjugated anti-rabbit or Alexa Fluor 488-conjugated anti-mouse antibodies) in 1% BSA. Fluorescence images were acquired and analyzed with a K1-Fluo confocal laser scanning microscope (Nanoscope Systems, Daejeon, South Korea). To quantify the degree of co-localization of proteins, fluorescence images were analyzed with ImageJ plug-in, JACoP (Just Another Co-localization Plugin), and Pearson’s correlation coefficient was calculated [22].

### Western blot

Total proteins from bEnd.3 cells or brain capillaries were extracted using RIPA buffer (Thermo Fisher Scientific) containing Halt Protease and Phosphatase Inhibitor Cocktail (Thermo Fisher Scientific). The protein concentration was determined using a Pierce™ BCA Protein Assay Kit (Thermo Fisher Scientific). Laemmli buffer containing 5% 2-mercaptoethanol was added to the protein samples, followed by heating at 95 °C for 3 min. Equal amounts of protein samples (30 μg/lane) were resolved using Tris–HCl sodium dodecyl sulfate-poly acrylamide gel electrophoresis (SDS-PAGE), and then transferred onto a PVDF membrane (Merck Millipore, Darmstadt, Germany). The membranes were blocked with 5% skim milk in Tris buffered saline with 0.1% Tween 20 (Bio-Rad, Hercules, CA, USA) (TBS-T) and then incubated with primary antibodies against LC3B, occludin, β-actin, p62/SQSTM1, or CD31 at 4 °C overnight. After washing with TBS-T, the membranes were incubated with the corresponding HRP-conjugated secondary antibodies. Protein bands were visualized by enhanced chemiluminescence using the Clarity Western ECL Substrate (Bio-Rad), SuperSignal West Pico Chemiluminescent Substrate (Thermo Fisher Scientific) or SuperSignal West Femto Maximum Sensitivity Substrate (Thermo Fisher Scientific). Relative levels of protein expression were quantified by NIH ImageJ software and normalized to the corresponding loading controls.

### Rat ischemic stroke model

For in vivo study, permanent ischemic stroke was induced in rats by intraluminal middle cerebral artery occlusion (MCAO). Experiments and procedures were performed in accordance with the institutional and international guidelines and regulations. All protocols for animal experiments were approved by the Institutional Animal Care and Use Committee (IACUC) at Hanyang University. Male Sprague–Dawley (SD) rats (230–300 g; Koatech, Gyeonggi, Korea) were housed in a specific-pathogen-free zone (12 h light/dark cycle, 23 °C, and 50% humidity). Rats were randomly divided into the treatment groups. To establish a rat ischemic stroke model, anesthesia was induced by isoflurane inhalation and maintained during the surgical period. Rectal temperature was monitored and maintained at 37 °C during surgery. The measurement of cerebral blood flow (CBF) was performed by laser Doppler (Perimed, North Royalton, OH, USA) before and after MCAO for 24 h. The left common carotid artery (CCA) and the external carotid artery (ECA) were carefully isolated and ligated tightly with a suture. After the ECA branch was cauterized, the internal carotid artery (ICA) was isolated and the pterygopalatine artery was coagulated. To initiate the ischemia, a silicone-coated 4-0 monofilament nylon suture (Doccol Co.) was inserted into the CCA. Approximately 18.0 mm of the suture was advanced through the ICA from the CCA bifurcation to reach the origin of the MCA [18]. The validity of the MCAO ischemic animal models was confirmed by histological analysis of brain sections stained with triphenyltetrazolium chloride (TTC; 2%), and the reduction of CBF in ischemic region. After occlusion, CBF decreased to less than 30% of baseline. For sham-operated rats, the CCA was only exposed to air.

To inhibit autophagic processes in the rats, 0.3 mg/kg of 3-MA in sterile saline was administered through a tail vein injection at 30 min prior to the initiation of MCAO. Rats in the sham-operation and MCAO groups were administered the same amount of sterile saline.

### Isolation of rat brain capillaries

Rats were transcardially perfused with saline and the brain was quickly isolated. The whole hemispheres were separated and homogenized with ice-cold physiological buffer containing 4 mM KCl, 147 mM NaCl, 3 mM CaCl_2_, 1.2 mM MgCl_2_, and 15 mM HEPES, pH 7.4. The brain homogenates were centrifuged at 3500×*g* for 10 min at 4 °C. The supernatant was discarded and the pellet was resuspended using 20% Ficoll (Sigma-Aldrich) in physiological buffer. The suspension was centrifuged at 25,000×*g* for 10 min at 4 °C. After removing the supernatant, the pellet was resuspended in 15% dextran (Sigma-Aldrich) and the suspension was carefully added to the same amount of 20% dextran. Then, the suspension was centrifuged at 25,000×*g* for 10 min at 4 °C. The supernatant was completely removed and the pellet was used for further western blot analysis. To validate the isolation of the brain capillaries from the brain homogenates, protein expression level of the molecular markers of endothelial cells and neuronal cells (CD31 and NeuN, respectively) was compared between the brain homogenates and isolated capillaries.

### Evans blue in vivo permeability assay

2% Evans blue (Sigma-Aldrich, USA) in sterile saline was injected into tail vein in rats (4 mL/kg) at 20 h after MCAO. After 4 h circulation, rat was transcardially perfused with saline and the brain was quickly removed. The brain hemisphere was homogenized in 2 mL of dimethylformamide (DMF) and incubated at 55 °C for 18 h, and then homogenates were centrifuged at 10,000×*g* for 20 min. The absorbance of supernatant was measured at 620 nm, using an EnSpire microplate reader (PerkinElmer).

### Statistical analysis

All experimental values were expressed as the mean and standard error (SEM). Statistical significance between groups was determined by the Student’s t-test. In all analyses, a *p* value < 0.05 was considered statistically significant.

## Results

### Cellular dysfunction and changes in occludin in brain endothelial cells following OGD stimulation

**Fig. 1:**
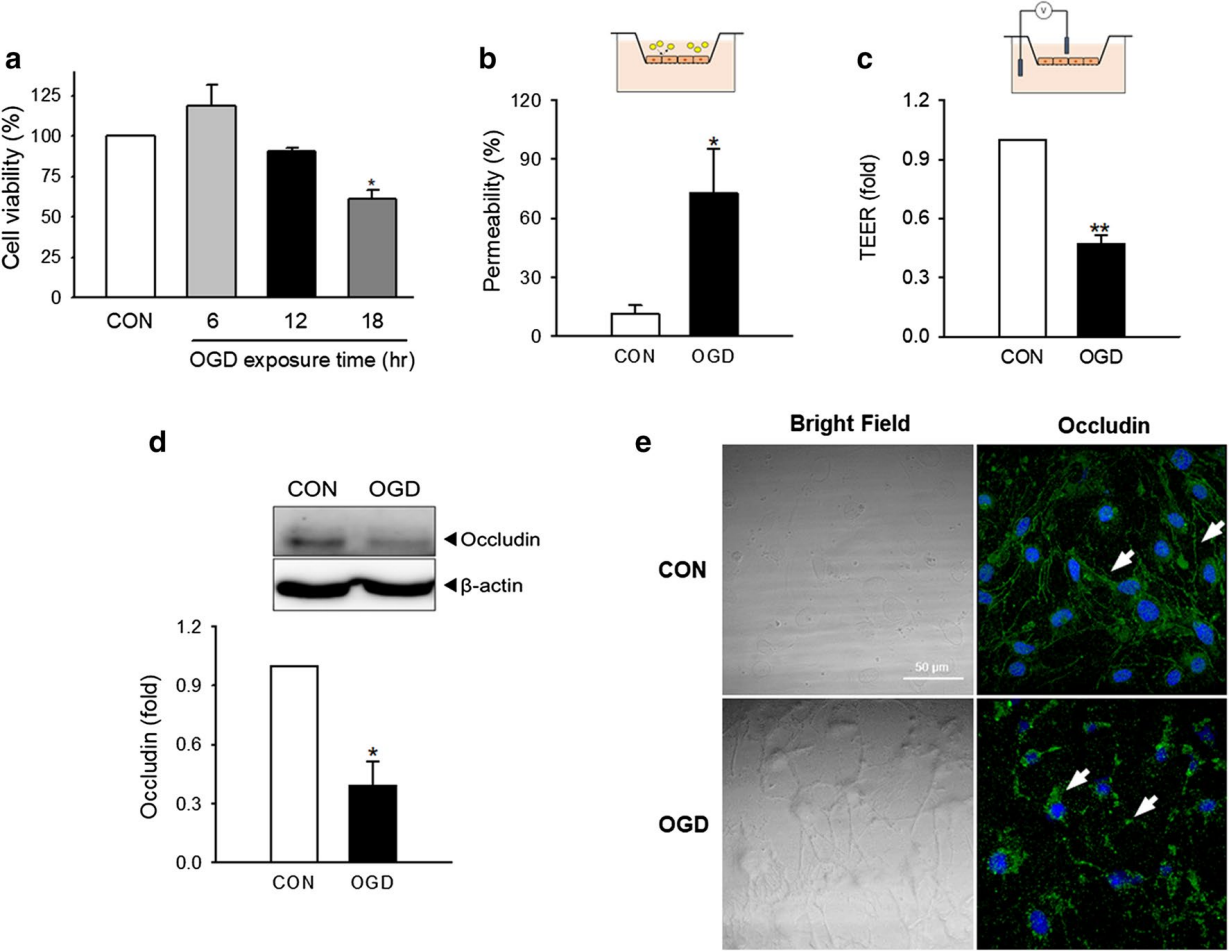
Changes on cell viability, permeability, and occludin in brain endothelial cells following exposure to oxygen–glucose deprivation (OGD). The brain endothelial cell line bEnd.3 was exposed to OGD for 6, 12 or 18 h. **a** Cell viability was examined by the MTT assay. N = 3–4. **b** Functional changes in cellular permeability were analyzed by an in vitro FITC-dextran permeability assay (N = 5) and **c** transendothelial electrical resistance (TEER) measurements (N = 3) after 18 h OGD. **d** The protein level of occludin after 18 h OGD was determined by western blot analysis. Relative occludin levels were normalized to β-actin. N = 4. **e** 18 h OGD-exposed bEnd.3 cells were stained with an antibody to occludin and visualized by immunofluorescence in confocal microscope. Representative images are shown. Scale bar: 50 μm. Data were expressed as the mean ± SEM and analyzed by the Student’s t-test. White arrows show occludin distribution. *p < 0.05 and **p < 0.01 vs. the control group without OGD

We used the bEnd.3 cell line as in vitro BBB model. To find an appropriate in vitro ischemic insult, cells were exposed to OGD for 6, 12 or 18 h to simulate ischemia and cell viability was determined. Cells exposed to 18 h OGD showed approximately 60% cell viability compared to control cells, suggesting that the ischemia decreased cell survival (Fig. 1a). To evaluate functional alteration by ischemic insult, bEnd.3 cells were exposed to OGD for 18 h and in vitro cellular permeability were measured (Fig. 1b, c). The relative cell permeability was determined by measuring the permeability of FITC-dextran across the cell monolayer and the transendothelial electrical resistance (TEER). The values of ischemic and control groups in both tests were calculated by normalizing to the cell-free membrane value. The permeability of cells exposed to OGD increased approximately sixfold compared to the control group (Fig. 1b) and the TEER value in OGD-exposed cells was also significantly decreased to about half of the control group (Fig. 1c)

Next, we examined whether OGD stimulation in bEnd.3 cells affected the level of occludin, an essential component of the tight junction proteins which is important for maintaining BBB integrity. Western blot results demonstrated a significant decrease in the amount of occludin protein in bEnd.3 cells exposed to OGD compared to controls (Fig. 1d). Intracellular distribution of occludin was examined using immunofluorescence staining. In OGD-exposed cells, occludin exhibited a dotted fluorescence signal only in the cytoplasm in OGD-exposed cells, suggesting that the intracellular distribution of occludin had shifted along with the decreased cellular occludin level upon ischemic insult (Fig. 1e). These results indicate that OGD triggers occludin degradation and redistribution in brain endothelial cells, accompanied by impaired permeability.

### Increased autophagy in OGD-exposed brain endothelial cells

**Fig. 2:**
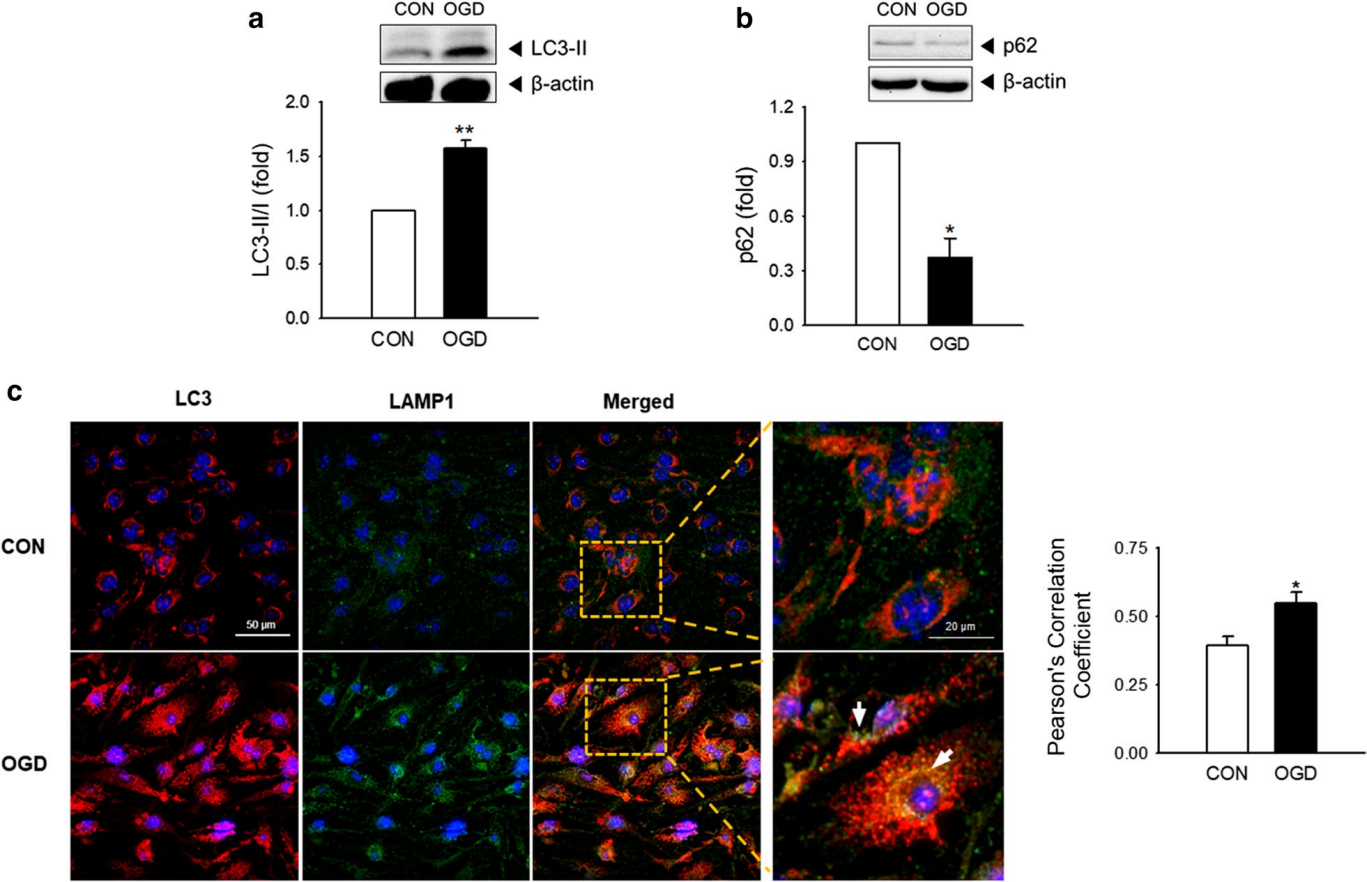
Activation of autophagy in OGD-exposed brain endothelial cells. The brain endothelial cell line bEnd.3 was exposed to OGD for 18 h. The protein levels of **a** LC3-II and **b** p62/SQSTM1 were determined by western blot. Protein levels were normalized to β-actin. N = 3. **c** Intracellular localization of LC3 and LAMP-1 was examined in OGD-exposed bEnd.3 cells using double immunostaining. Representative images are shown. Scale bar: 50 μm, scale bar in the magnified images: 20 μm. White arrows show the co-localization of LC3 and LAMP1. Pearson’s Correlation Coefficient were calculated using JACoP. Data are presented as the mean ± SEM and analyzed by the Student’s t-test. *p < 0.05 vs. control

To investigate whether autophagy is activated in bEnd.3 cells after OGD, we examined changes in the protein levels of the well-established autophagy indicators, LC3-II and p62/SQSTM1 (Fig. 2a, b). The level of LC3-II correlates with the number of autophagosomes during autophagy. The adapter protein p62/SQSTM1 binds to the LC3 on the autophagosome membrane and is degraded by the fusion of the autophagosomes with lysosomes [23]. Relative to controls, the amount of LC3-II was increased approximately sevenfold, indicating that autophagosome formation was increased in OGD-exposed bEnd.3 cells (Fig. 2a). In contrast, the amount of p62 was reduced by approximately 0.4-fold, demonstrating that autophagic flux was activated in the cells by OGD exposure (Fig. 2b). While LC3 is spread evenly within normal cells, it is observed as punctate aggregates throughout cells when autophagy is activated. To further investigate autophagosome formation and autophagic flux, the intracellular location of LC3 (autophagosomal marker) and LAMP1 (lysosomal marker) were determined by orange and green immunofluorescence staining, respectively. Both of the fluorescence signals were randomly and evenly distributed in control cells. However, in OGD-exposed cells, fluorescent orange puncta were observed along with co-localized green signals, demonstrating the fusion of autophagosomes with lysosomes (Fig. 2c). These data suggest that OGD exposure activates autophagic pathways and autophagic flux in brain endothelial cells.

### Occludin as a potential target protein of autophagic degradation in OGD-exposed brain endothelial cells

**Fig. 3:**
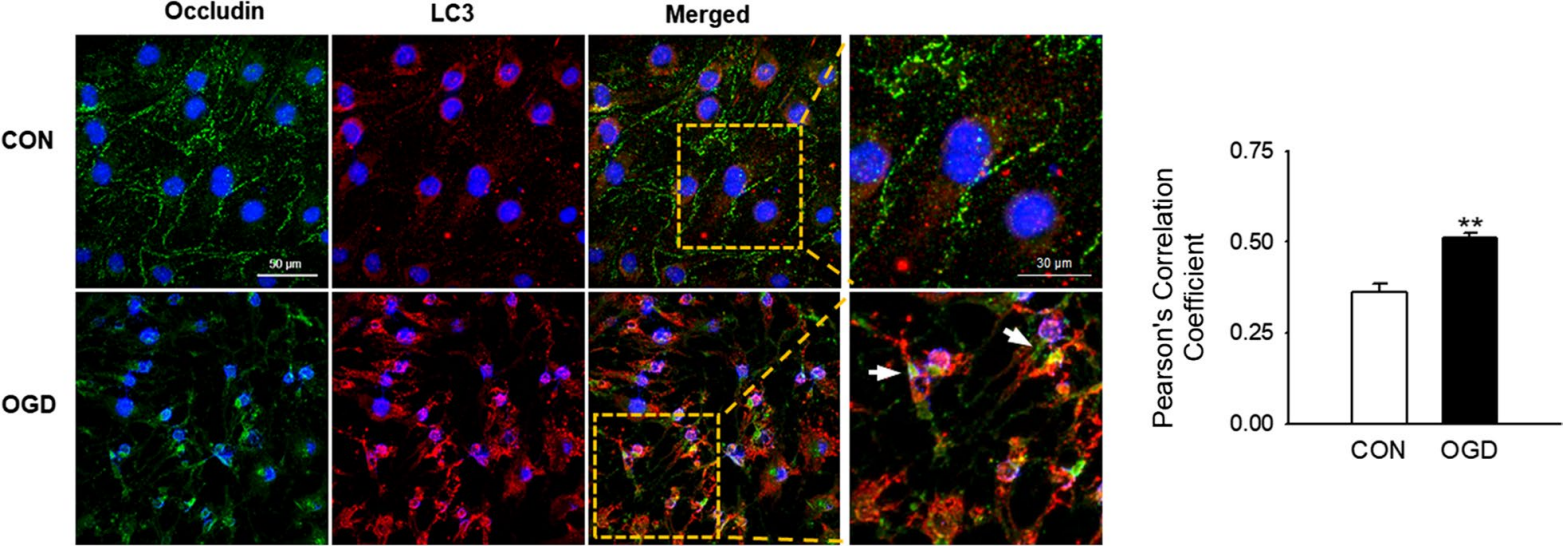
Co-localization of LC3 and occludin in OGD-exposed brain endothelial cells. After exposure of bEnd.3 cells to OGD for 18 h, cells were stained for occludin and LC3 by immunofluorescence. Representative images are shown. Scale bar: 50 μm, scale bar in the magnified images: 30 μm. White arrows show the co-localization of occludin and LC3. Pearson’s Correlation Coefficient were calculated using JACoP. Data are presented as the mean ± SEM and analyzed by the Student’s t-test. **p < 0.01 vs. control

Next, we investigated whether the changes in occludin levels and localization following OGD exposure are due to autophagy. Double immunofluorescence staining for occludin and LC3 was performed to examine the association between OGD-induced autophagy and occludin degradation. In control cells, the fluorescence signals of LC3 and occludin were evenly dispersed, while both proteins were observed as punctate aggregates in cells exposed to OGD (Fig. 3). Furthermore, occludin and LC3-positive puncta were co-localized in cells after OGD exposure (Fig. 3), indicating that autophagy is a possible degradative pathway of occludin in brain endothelial cells during OGD.

### Restoration of OGD-induced cell damage through the inhibition of autophagy by 3-MA in brain endothelial cells

**Fig. 4:**
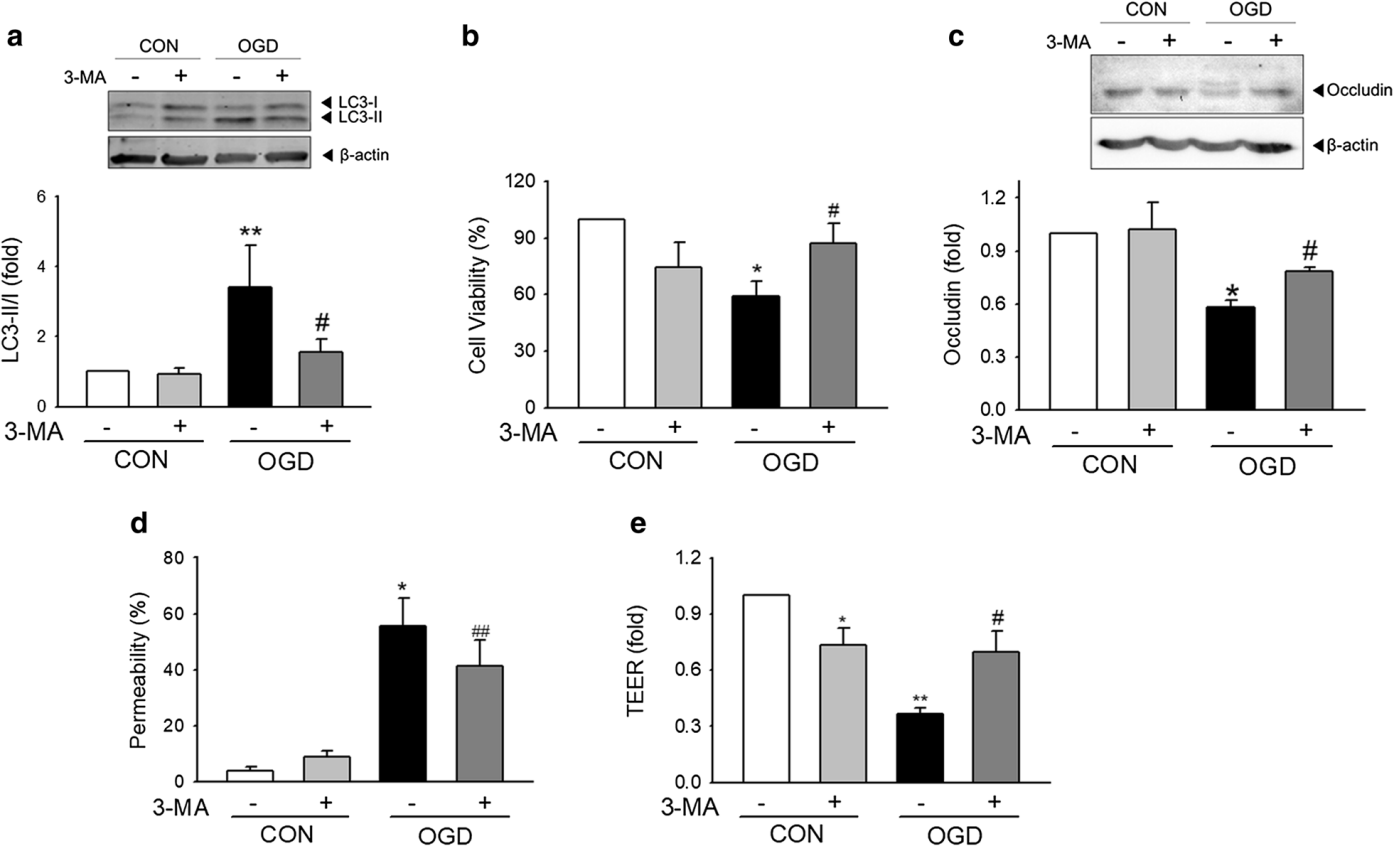
Role of ischemic autophagy in OGD-induced dysfunction in brain endothelial cells. bEnd.3 cells were treated with 3-MA for 1 h prior to and during the 18 h OGD exposure. **a** The protein amount of LC3-II was determined by western blot. β-actin was used as a loading control. N = 7. **b** Changes in cell viability were analyzed by the MTT assay. N = 3. **c** The protein levels of occludin were detected by western blot. The bands were normalized by β-actin levels. N = 3. **d** In vitro FITC-dextran assay and **e** TEER measurement were conducted to examine cellular permeability. N = 4. Data are presented as the mean ± SEM. *p < 0.05 and **p < 0.01 vs. control; ^#^*p *< 0.05 and ^##^*p *< 0.01 vs. OGD without 3-MA

We next investigated whether OGD-induced cellular dysfunction and occludin degradation are restored by inhibiting autophagy by 3-MA, a well-established autophagy inhibitor [24, 25]. Before and during OGD exposure, cells were incubated in the presence or absence of 5 mM of 3-MA. LC3-II levels were increased by approximately sixfold in OGD-exposed cells compared to controls, but this increase was significantly attenuated through the inhibition of autophagy by 3-MA (Fig. 4a), confirming that 3-MA (5 mM) treatment inhibits OGD-triggered autophagy in bEnd.3 cells. Next, we examined whether autophagy inhibition by 3-MA restores OGD-induced cellular dysfunction. Cells exposed to OGD showed a significant decrease in cell viability, which was significantly restored by autophagy inhibition with 3-MA (Fig. 4b). Moreover, autophagy inhibition by 3-MA treatment prevented the OGD-induced degradation of occludin (Fig. 4c), resulting in attenuation of the OGD-impaired cellular permeability (Fig. 4d, e). These results demonstrate that autophagy contributes to change of cell viability, decrease in occludin level, and abnormal hyper-permeability induced by OGD in brain endothelial cells.

### Ischemia-induced autophagy and occludin degradation in vivo

**Fig. 5:**
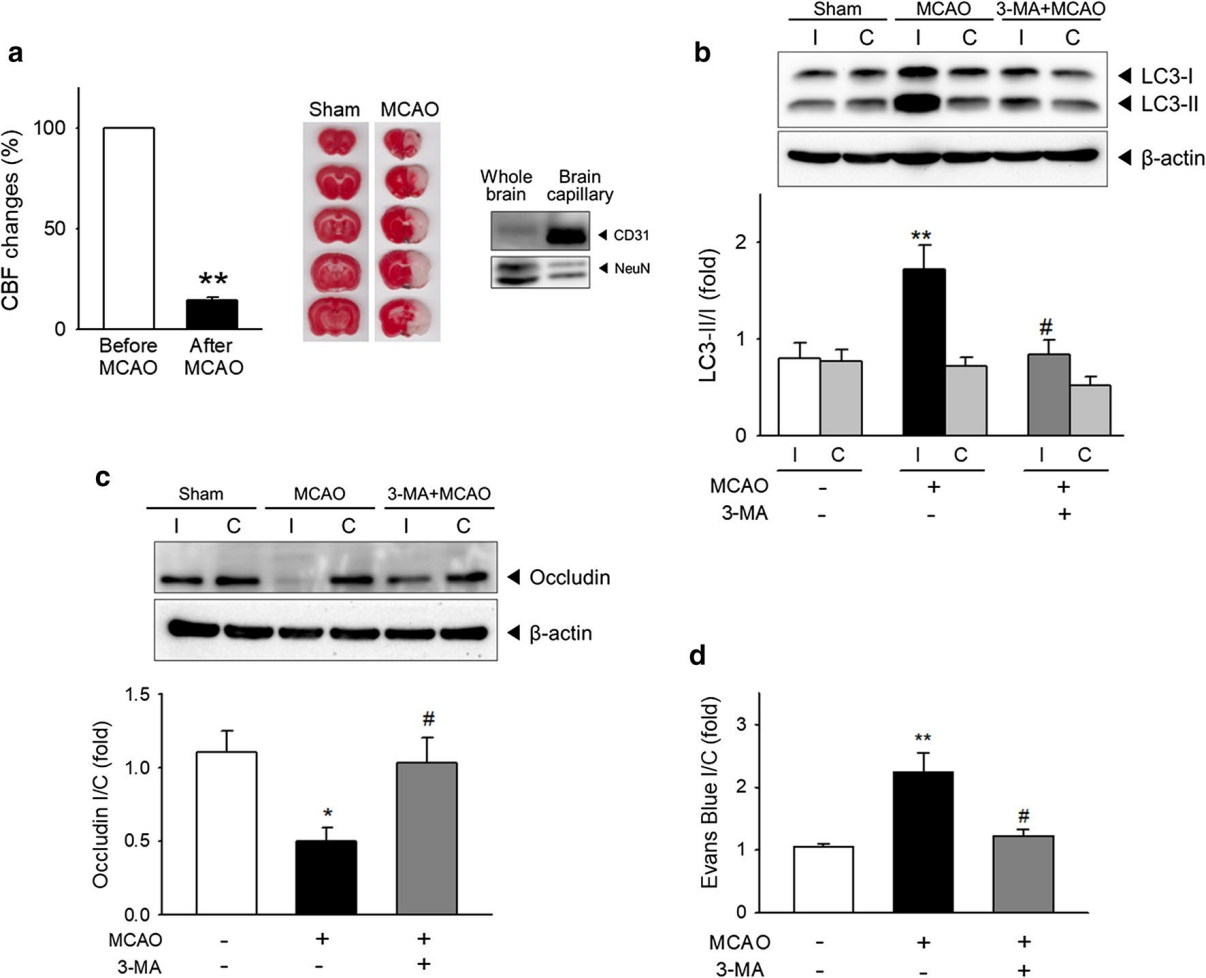
Autophagy and occludin degradation in brain capillaries from rats with ischemic stroke by MCAO. **a**–**c** Ischemic stroke was induced in rats by middle cerebral artery occlusion (MCAO). At 24 h after MCAO, rats were sacrificed by transcardial perfusion, and brain capillaries were isolated from both the ischemic and non-ischemic hemispheres. 3-MA (0.3 mg/kg) or sterile saline solution was administrated to rats at 30 min prior to MCAO. N = 5–9. **a** Cerebral blood flow (CBF) changes were monitored before and after MCAO (Right). TTC-stained brain sections confirmed the ischemic brain damage (Middle). The protein levels of endothelial and neuronal marker (CD31 and NeuN, respectively) in the whole brain homogenates and isolated brain capillary were measured (Left). **b** Autophagic activation in brain capillaries was examined based on the level of LC3-II. The conversion ratio of LC3-II/I was calculated. **c** The amount of occludin protein in the brain capillaries was detected by western blot. Protein levels were normalized with the corresponding β-actin levels. **d** 2% Evans blue (4 mL/kg) was intravenously injected at 20 h after MCAO. The rats were transcardially perfused to remove any remaining Evans blue in blood and sacrificed at 24 h after MCAO. The amount of Evans blue was determined in ipsilateral or contralateral hemisphere. N = 5–7. Data are expressed as the mean ± SEM. *p < 0.05 and **p < 0.01 vs. sham-operated rats, ^#^*p *< 0.05 vs. rats with MCAO without 3-MA treatment

We used rats with MCAO for a permanent ischemic stroke model to examine whether autophagy is involved in in vivo BBB dysfunction in cerebral ischemia. To confirm the ischemic insult in rats, the cerebral blood flow (CBF) changes suture was measured before and after intraluminal MCAO (Fig. 5a left), and the brain sections were stained with TTC (Fig. 5a middle). After isolation of brain capillaries from rats following ischemia, the protein expression levels of endothelial marker (CD31) and neuronal marker (Neu N) were compared (Fig. 5a right), to make sure if the separated fraction is mainly composed with endothelial cells. To determine whether autophagy is induced in the BBB during cerebral ischemia, the protein levels of LC3-I and LC3-II in brain capillaries were quantified from both the ischemia-injured (ipsilateral) and non-injured (contralateral) hemispheres. LC3-I conversion to LC3-II was significantly enhanced in ipsilateral brain capillaries isolated from MCAO-operated rats compared to that in contralateral brain capillaries or in both hemispheres from the sham-operated rats (Fig. 5b). MCAO-induced LC3 conversion in ipsilateral brain capillaries was significantly inhibited by intravenous administration of 3-MA to levels similar to that of sham-operated rats (Fig. 5b). These findings indicate that autophagy is activated in the BBB during ischemic stroke and is inhibited by intravenous 3-MA administration (0.3 mg/kg).

Next, we examined occludin levels in brain capillaries to investigate whether ischemia-induced autophagy contributes to alterations in TJ proteins (Fig. 5c). The ipsilateral-to-contralateral (I/C) ratio of occludin was decreased in MCAO-operated rats to approximately half that of sham-operated rats. 3-MA administration restored the decrease in the occludin I/C ratio in MCAO rats, indicating that autophagy mediates the degradation of occludin during ischemia. These in vivo results using brain capillaries are consistent with our in vitro data. Taken together, our data from both in vitro and in vivo models demonstrate that autophagy is an important pathway responsible for occludin degradation during cerebral ischemia.

To further evaluate the effect of autophagy inhibition by 3-MA on ischemic BBB function in vivo, the integrity of BBB was determined using Evans blue leakage in rats. The extravasation of intravenously administered Evans blue to brain tissue indicates the disruption of BBB. While I/C ratio of Evans blue in MCAO-operated rats was increased following MCAO suggesting that ischemic injury increased BBB permeability, administration of 3-MA significantly reduced the increased I/C ratio of Evans blue (Fig. 5d).

## Discussion

When blood flow to the brain is interrupted during ischemic stroke, which is one of the leading causes of death and disability [26], the depletion of oxygen and nutrients in the ischemic area triggers a cascade of detrimental processes leading to neuronal cell death, neuro-inflammation, and cerebrovascular disruption [15, 27–29]. There have been extensive attempts to understand the pathophysiology of ischemic stroke [18, 28, 30–32]. Recently, the concept of the neurovascular unit (NVU) has been used to explain the pathophysiology of brain diseases including stroke. In the NVU, the brain is described as an integrated system with diverse cellular components, and harmonized interactions between these cells are crucial for normal brain function [33]. Of note, brain endothelial cells are the main component of the NVU. Compromise of the NVU during ischemia results in BBB disruption and the influx of inflammatory cells and substances from the systemic circulation which amplifies brain injury [34]. The elucidation the molecular mechanisms underlying ischemic BBB damage is pivotal to understand NVU impairment during ischemia.

Previous studies have focused on MMP-mediated degradation of TJs in brain endothelial cells [35, 36]. However, the precise mechanisms and pathways that ultimately lead to BBB dysfunction are largely unknown. The findings of our study show that autophagy, which plays a role in cellular maintenance in normal cells [37], might also play a role in damaging cellular components during pathological processes such as stroke. Our results demonstrated that ischemia-induced autophagy mediated the degradation of occludin and that inhibition of autophagy by 3-MA reversed occludin degradation and attenuated BBB dysfunction. In line with our observation, claudin-5 was degraded by autophagy-lysosome activation in OGD-exposed brain endothelial cells [38], and autophagic activation with BBB damage were observed in p50 knockout mice [39], suggesting that autophagy may contribute to ischemic neuronal and vascular damage. It is also demonstrated that enhanced autophagy during brain ischemia in diabetic mice might be responsible for excessive BBB disruption [40].

Contrary to our findings, Li et al. recently reported the protective potential of autophagy in brain endothelial cells and BBB dysfunction during cerebral ischemia/reperfusion (I/R) injury [41]. In their study, activation of autophagic processes using the autophagy inducers rapamycin and lithium carbonate significantly reversed BBB disruption following I/R injury, while inhibition of autophagy with 3-MA pretreatment intensified I/R-induced BBB damage. Although the specific cellular target of activated autophagy was not identified in their study, the authors concluded that autophagy protected cells against the generation of reactive oxygen species and restored the decreased level in ZO-1 levels. There are differences between the experimental models used in our study and those used by Li et al. While we used 18-h OGD in cells and 24-h MCAO in rats, they used 6-h OGD/4-h reoxygenation in cells and 2-h ischemia/22-h reperfusion in rats. Supporting our findings, a recent study by Liu et al. demonstrated that another BBB component claudin-5 is degraded by 4-h OGD, but not by 2-h OGD/2-h reoxygenation, and suggested that the degradation of claudin-5 is mediated by caveolin-1 and autophagy-lysosome activation in endothelial cells [38]. However, in vivo experiments were not performed in the study by Liu et al. We believe that these previous observations along with our current findings contribute to an integrated understanding of the role of autophagy in ischemic BBB dysfunction.

Still, our current study has limitations in terms of the lack of evidence for ischemic duration-dependent contribution of autophagy in occludin change and BBB integrity during dynamics of ischemic injury. The duration of 18 h for OGD stimulation induced cell death (Fig. 1a) and the decreased cell viability itself can possibly contribute to the increased permeability (Fig. 1b, c), not essentially mediated by autophagy-mediated occludin degradation. Although we could not exclude the cell death itself can contribute to functional alteration, the decrease of occludin protein is not non-specific phenomena accompanied by cell death. Unlike occludin, the total level of ZO-1, another important TJ protein, was not affected by 18 h OGD in our system (data not shown). Moreover, the autophagic inhibition by 3-MA reversed autophagy, occludin degradation, cellular permeability and cell death, suggesting that at least autophagy may be an important mediator of these consecutive phenomena (Fig. 4). The relationship between ischemic insult and the TJ protein expression along with the role of autophagy should be examined according to the ischemic duration also with the presence of reperfusion in the future study, to comprehensively understand the dynamics of ischemic BBB injury.

It is known that moderate autophagy plays a protective role against pathological conditions since it rapidly removes damaged cellular components which contribute to cellular dysfunction, but excessive autophagy itself can target the normal pivotal components leading to cellular damage and aggravation of the pathological processes [42–44]. The opposing roles of autophagy have been reported in diverse pathological conditions such as stroke, diabetes, and several neurodegenerative diseases, suggesting that autophagy is a delicate regulator for cell function, ultimately affecting cell survival and death [44–46]. Here, we have shown that occludin is a direct target of ischemic autophagy resulting in ischemic BBB dysfunction. However, the determination of the precise role of autophagy in each component of the BBB and its relative contribution will require further investigation. This includes the extent and time course of autophagic activation as well as the substances targeted for degradation in the absence or presence of reperfusion during ischemic stroke. It would be also worthy to elucidate if non-ischemic autophagic activation, such as pharmacologically induced by rapamycin, or pathologically induced by starvation, etc., could be involved in occludin degradation in brain endothelial cells and BBB integrity.

We have also shown that the TJ protein occludin is degraded by cytoplasmic autolysosomes, which is an important insight into the potential role of autophagy in degradation of the transmembrane cytoskeleton, suggesting that the target substrates of autophagic degradation are not limited to intracellular components. There may be intracellular trafficking signaling for the translocation of TJ proteins, which is essential for degradation of these proteins by autolysosomes. Liu et al. suggested that nitric oxide (NO) facilitated caveolin-1-mediated translocation of claudin-5 to autophagosomes in endothelial cells [38]. The molecular mechanisms that mediate occludin modification and translocation to autolysosomes during autophagy-mediated degradation during ischemia remain to be elucidated. Another interesting point is that the tight junction proteins in BBB contains oligomeric protein assembly which is pivotal for maintaining BBB integrity [47]. TJ transmembrane protein homodimers including occludin multimers are essential in TJ complex assembly. In this study, we have measured the alteration of occludin monomer (~ 52 kDa) by ischemic damage in bEND.3 cells, which was reversed by autophagic inhibition. Further studies warrant the autophagic regulation of TJ protein multimers to clarify the role of autophagy in TJ proteins and BBB integrity.

We used levels of LC3-II as a representative marker for autophagic activation. An increase in the LC3-II protein levels, which indicates an increase in autophagosomes does not always indicate normal autophagy flux. In several pathological conditions, the accumulation of autophagic vacuoles and the corresponding increase in LC3-II were often observed due to a failure of fusion between autophagosomes with lysosomes, and these phenomena were explained as abnormal autophagy rather than autophagic activation [48, 49]. We examined the formation of autolysosomes by co-localization of intracellular LAMP1, a lysosomal marker, and LC3 in order to elucidate whether increased LC3-II indicates abnormal autophagy. LC3 and LAMP1 proteins showed a co-localized intracellular distribution under OGD stimuli, indicating that autophagy had occurred rather than LC3-II protein accumulation by abnormal autophagy. In addition, the level of protein p62, an autophagy adapter protein, was decreased by OGD in endothelial cells, further supporting the occurrence of autophagy.

## Conclusion

There has been accumulating evidence that autophagy plays important roles in ischemic BBB damage, but the exact contribution of autophagy or the target autophagic cargo still remains unclear [50]. Here, we have demonstrated that ischemia-induced autophagy occurs in brain endothelial cells in vitro and rat brain capillaries in vivo and that this mediates the degradation of occludin, a major component of TJs in the BBB. Furthermore, we showed that ischemic autophagy increased brain endothelial permeability which can be attenuated by inhibiting autophagy. Taken together, our current findings contribute to an integrated understanding of the role of autophagy in ischemic BBB dysfunction.

## Data Availability

The datasets used and/or analyzed during the current study are available from the corresponding author on reasonable request.

## Abbreviations

BBB: Blood–brain barrier
OGD: Oxygen glucose deprivation
NVU: Neurovascular unit
3-MA: 3-Methyladenine
MCAO: Middle cerebral artery occlusion
ZOs: Zonula occludens
LAMP-1: Lysosomal-associated membrane protein 1
TEER: Transendothelial electrical resistance
CBF: Cerebral blood flow
CCA: Common carotid artery
ECA: External carotid artery
ICA: The internal carotid artery
I/C: Ipsilateral-to-contralateral
